# Efficacy of a computer based discontinuation strategy to reduce PPI prescriptions: a multicenter cluster-randomized controlled trial

**DOI:** 10.1038/s41598-023-48839-2

**Published:** 2023-12-07

**Authors:** Julia Heisig, Bettina Bücker, Alexandra Schmidt, Anne-Lisa Heye, Anja Rieckert, Susanne Löscher, Oliver Hirsch, Norbert Donner-Banzhoff, Stefan Wilm, Anne Barzel, Annette Becker, Annika Viniol

**Affiliations:** 1 Department of Primary Care, University of Marburg, Marburg, Germany; 2 Institute of General Practice (Ifam), Centre for Health and Society (Chs), Medical Faculty, Heinrich Heine University Düsseldorf, Düsseldorf, Germany; 3 Chair of General Practice II and Patient-Centeredness in Primary Care, Institute of General Practice and Primary Care (IGPPC), Faculty of Health, Witten/Herdecke University, Witten, Germany; 4 Chair of General Practice I and Interprofessional Care, Institute of General Practice and Primary Care (IGPPC), Faculty of Health, Witten/Herdecke University, Witten, Germany; 5 Department of Psychology, FOM University of Applied Sciences, Siegen, Germany; 6 Department of General Practice and Primary Care, Ulm University Hospital, Ulm, Germany

**Keywords:** Gastroenterology, Medical research

## Abstract

Deprescribing of inappropriate long-term proton pump inhibitors (PPI) is challenging and there is a lack of useful methods for general practitioners to tackle this. The objective of this randomized controlled trial was to evaluate the effectiveness of the electronic decision aid tool arriba-PPI on reduction of long-term PPI intake. Participants (64.5 ± 12.9 years; 54.4% women) with a PPI intake of at least 6 months were randomized to receive either consultation with arriba-PPI from their general practitioner (n = 1256) or treatment as usual (n = 1131). PPI prescriptions were monitored 6 months before, 6 and 12 months after study initiation. In 49.2% of the consultations with arriba-PPI, the general practitioners and their patients made the decision to reduce or discontinue PPI intake. At 6 months, there was a significant reduction by 22.3% (95% CI 18.55 to 25.98; p < 0.0001) of defined daily doses (DDD) of PPI. A reduction of 3.3% (95% CI − 7.18 to + 0.62) was observed in the control group. At 12 months, the reduction of DDD-PPI remained stable in intervention patients (+ 3.5%, 95% CI − 0.99 to + 8.03), whereas control patients showed a reduction of DDD-PPI (− 10.2%, 95% CI − 6.01 to − 14.33). Consultation with arriba-PPI led to reduced prescription rates of PPI in primary care practices. Arriba-PPI can be a helpful tool for general practitioners to start a conversation with their patients about risks of long-term PPI intake, reduction or deprescribing unnecessary PPI medication.

## Introduction

Proton pump inhibitors (PPI) are indicated to treat gastritis, peptic ulcer disease, symptomatic gastroesophageal reflux disease (GERD) and acid-related complications (e.g. erosive esophagitis). It is recommended in international guidelines to discontinue PPI after healing of uncomplicated GERD^1,2^, gastritis, gastroduodenal ulcer disease and *H. pylori* eradication therapy^3^. Long-term PPI intake is indicated in patients with symptomatic GERD, Barrett’s esophagus or who are at high risk for ulcer-related bleeding due to prolonged therapy with nonsteroidal anti-inflammatory drugs (NSAID) or antiplatelet agents. The dose of long-term PPI should be reevaluated regularly with the goal to prescribe the lowest effective dose^4^. Prolonged PPI intake can be a risk factor for enteric infection, cardiovascular disease and heart failure or malabsorption of nutrients, minerals and vitamins^5–7^. On the one hand, PPI is a relatively well-tolerated drug for many patients, and nearly all adverse outcomes associated with PPI occur among patients who receive long-term therapy^6^. On the other hand, it contributes to polypharmacy and is therefore a good candidate for deprescribing.

In the primary care practice, many patients are prescribed PPI for symptomatic control of uncomplicated gastritis or GERD, as well as for stress ulcer prophylaxis after hospital release^8,9^. Contrary to guideline recommendations, a large number of these patients take PPIs long-term and PPI prescription numbers have been increasing for years^10–13^. The reasons for this are complex: lack of critical re-evaluation in follow-up prescription situations, lack of awareness of potential harmful effects of PPIs, low therapy costs, and even discontinuation symptoms after prolonged use^14–16^. How can a general practitioner identify a case of PPI overuse and approach deprescribing?

Publication of a new NIH guideline in 2014 did not change prescribing patterns^17^. There are interventions in primary care and hospital settings aiming to identify and discontinue inappropriate use of PPI with successful results^18–20^. Despite these and several more studies on deprescribing PPI^4,21,22^, there is no established method that is regularly used in general practice. General practitioners who are aware of proper indications for PPI use and the perception or hospital physician’s competence (and also less financial pressure in hospitals, therefore higher readiness to prescribe medication) are more eager to quit inappropriate medication^14^.

Arriba is a computer software that supports shared-decision making on different topics^23^. It is available since 2010 and is regularly used in many German family practices (e.g. the module for cardiovascular prevention, arriba-KVP). Thus, arriba-PPI as a new module on the known platform has the possibility to reach a higher number of physicians. This module was developed to reduce the overutilization of PPI by including both views of physician and patient to reach a shared decision-making. How does it work? In a first step, the reasons for the patients’ PPI intake are discussed considering current and earlier experienced complaints, followed by the question whether the indication for PPI is still given. The programme provides information on the evidence for the use of PPIs to support the physician and the patient in their decision on proceeding PPI. In situations where discontinuation is potentially possible, the programme also facilitates individual decision-making by presenting the advantages and disadvantages of discontinuation. These serve as a basis for discussion between the doctor and the patient. Patients who ultimately decide to discontinue are given advice on how to reduce the dose and how to deal with potentially arising rebound problems.

The aim of this study was to investigate the effectiveness of the computer-based consultation aid arriba-PPI on the rate of PPI prescriptions in general practice. Our hypothesis was that the electronic decision aid arriba-PPI reduces PPI prescriptions (cumulative defined daily doses (DDD) per patient) in general practices about at least 20% more than a conventional consultation.

## Methods

### Trial design

This study is a multicenter, cluster-randomized controlled trial with two evenly randomized arms. A cluster is defined as the total recruited patients per praxis. Data were collected at three timepoints: pre-intervention (baseline, T0), at 6 months (T1) and 12 months (T2). The study was conducted in general practices in the German regions of Middle- and North-Hessen and Ruhr and Rhine valley in North Rhine-Westphalia. Three study centers were involved: Department of General Practice of Marburg University; Institute of General Practice of Heinrich-Heine-University Düsseldorf; and Institute of General Practice and Primary Care of Witten/Herdecke University. The study protocol was published by Rieckert et al.^24^. The study was registered retrospectively on 31.01.2019 at the German Clinical Trial Register (DRKS00016364).

### Ethics approval

The study was approved by three local ethics committees (Marburg University, 20.08.2018, ref. 73/18; Witten/Herdecke University, 18.09.2018, ref. 146/2018; and Heinrich-Heine-University Düsseldorf, 03.12.2018, ref. 2018–249). We confirm that written informed consent was obtained from all participants and/or their legal guardians. Research has been performed in accordance with the Declaration of Helsinki.

### Participants

Inclusion criteria for general practitioners (GPs) were German as the main language in patient care, technical requirements for using a computer based tool in the consultation room, willingness to share PPI prescription data from their practice software and consent to collect data about PPI prescription data by the German health insurance company AOK. Excluded were physicians with specialized focus without regular PPI prescriptions (e.g. psychological therapy or acupuncture practices) or without a computing management system.

Inclusion criteria for patients comprised age ≥ 18 years and regular PPI prescription for at least 6 months. Definition of long-term use varies widely in the literature (most common are ≥ 8 weeks up to 1 year), depending on outcome measurements of side effects or initial diagnosis, among others^25,26^. In the context of our study, we define an intake as "long-term" if it lasts ≥ 6 months. According to the guideline, after this time the healing periods for the most common indications should be completed and a reassessment should have taken place^27^.

Not included were patients with poor German language skills, cognitive impairments that hinder a study information or consent and housebound patients (e.g. patients with fragilities that impede a personal visit to the physician’s practice).

### Recruitment

Primary care physicians’ offices in the German regions described above were invited to participate by phone call or letter/e-mail including a fax response with their contact details to be called back. A study team member visited all interested practices to hand over questionnaires for data collection. Informed consent was obtained from all the participants. In participating practices, medical assistants were instructed to recruit, within the following 6 weeks, 15–20 study patients who came to the counter to refill their PPI prescriptions in a consecutive manner. After completion of recruitment, the practices sent the patient recruitment lists to their respective study center per mail or fax. Patient recruitment was staggered from November 2018 to July 2019, with the subsequent data assessment period extending from December 2018 to October 2020. After patient recruitment, the practices were randomized.

### Complex intervention

The intervention for the patients was delivered at the level of general practice and was prepared by members of the universities’ study teams as follows. After randomization, study team members visited each practice from the intervention group to install the arriba-PPI software on a computer in the treatment room. The GP was then trained in discontinuation strategies, shared decision making and how to use arriba-PPI with a 10 min video in German language (https://arriba-hausarzt.de/module/ppi-protonenpumpen-hemmer-absetzen). The day of training in the intervention group was defined as T0. Within the following 6 weeks, study patients were scheduled for an appointment with their GP to receive consultation with arriba-PPI. For further care and until end of study, the GPs were instructed to treat their study patients as necessary and as usual for 12 months.

### Control

After randomization, control practices were directed by phone to treat their study patients as usual for 12 months. These practices did not make any extra appointments with their patients for this study. The day of the phone call in the control group was defined as T0.

### Telephone interview

All patients of the intervention and control group were interviewed by telephone at T1 to gain information about their current PPI medication and the reasons for taking it.

### Data collection

Baseline characteristics of GPs and patients were collected with questionnaires and recruitment lists provided during study personnel visits in the practices. PPI prescription data (agent, dose, package size, prescription date) were obtained retrospectively from the practice software in each general practice for a time span of 6 months before T0 to T2 (1.5 years). These data were collected during visits of study personnel in each practice or after instructions by phone and delivery per mail, E-mail or fax, depending on the preferences of practice personnel.

Collected PPI data was analyzed according to Germany’s WIdO ATC/DDD methodology that defines medication doses typically used in the main indication in adults per day^28^. The DDD is a measure of the amount of drug prescribed. It is calculated as follows (using pantoprazole as an example): the number of tablets prescribed (e.g. 100 tablets) is multiplied by the prescribed dose of the respective tablets (e.g. 40 mg), and divided by the dose typically used in the main indication in adults per day (for pantoprazole, for example, this corresponds to 20 mg) which results in 200 DDD. According to this procedure, DDD were calculated for each patient for a time span of 6 months at T0, T1 and T2.

### Outcomes

Primary outcome was the comparison of cumulated defined daily doses (DDD) of PPI per study patient after 6 months in the intervention and control group (T1). Secondary outcomes were the cumulated DDDs of PPI per study patient after 12 months (T2) and PPI intake status over time.

### Sample size

Sample size calculation was described in Rieckert et al.^24^, but was changed due to lower recruitment success. Preliminary results led to the assumption that a 20% reduction in DDD PPI compared to control could be achieved (instead of 15% reduction as originally hypothesized). Assuming an ICC of 0.07 and a significance level of 0.05, a power of 80% can be achieved with 94 practices of 15 patients each (instead of an ICC of 0.1 and 204 practices).

### Randomization

The GP practice was regarded as the unit of randomization. A simple randomization scheme was generated by the random package of the programme R. An independent trusted person outside the study team at the universities assigned practices (and their patients) to intervention or control group according to the randomization sequence. To assure concealment of allocation, no patient was included once recruitment was completed and randomization was performed.

### Blinding

Due to the nature of the intervention, neither GPs nor patients were blinded. For practical reasons, study personnel were not blinded either. However, a blinded statistician conducted all analyses.

### Statistical methods

Categorical variables regarding demographic characteristics of the general practices and participating patients were analysed using the chi-square test and the corresponding effect size Cramér V. Values < 0.20 signal a small effect, between 0.21 and 0.39 there is a moderate effect, and values > 0.40 signal a strong effect^29^. If there were more than 25% of cells in contingency tables with expected frequencies less than 5, Fisher´s Exact Test was used. For metric data the t-test was performed. Cohen’s d was used as an effect size, with a value of 0.2 to 0.49 representing a small effect, a value of 0.5 to 0.79 representing a medium effect, and a value of 0.8 and higher representing a large effect^29^.

#### Multilevel multiple imputation

For the main outcome variables of the sum of DDD of PPI, there were 0.7% missing values at time T0, 2.8% missing values at time T1, and 10.8% missing values at time T2, with these occurring exclusively in the intervention group at time T0 and similar distribution between the two groups at the other time points. Multilevel multiple imputation was performed using the R package mice 3.11.0 and method 2l.norm, which uses the linear mixed model with heterogeneous error variance^30^. The model included the additional variables age, gender, number of prescriptions at T0, T1 and T2. For the latter, missing data were replaced as well. The same proportions of missing values existed as for the DDD-PPI sum variables. Inclusion of additional variables did not result in convergence of the model. Twenty data sets each were imputed separately for the intervention and control groups, and the resulting objects were merged for subsequent analyses. The robustness of the results was tested by a complete case analysis^31,32^.

#### Multilevel modeling

The primary outcome was evaluated using multilevel analyses within Programme Package R^33^. These take into account the clustering of patients in practices and allow for different modeling with respect to predictors, e.g. group membership as fixed and/or random effect. An intention-to-treat analysis was applied.

Norman advocates an ANCOVA design in which correction is made for the baseline value and regression is performed on the outcome. This would also take into account the regression to the mean, since individuals with extreme values at the beginning of a study would tend to spontaneously converge to the mean of the respective sample^34^. The use of percentage change values related to a baseline is criticized in several publications. It is stated that the calculation of percent change values is statistically inefficient, and it is argued that they should not be used, but instead a covariance analysis approach (ANCOVA) with the baseline value as covariate. Various simulations were calculated for different correlations between baseline and follow-up scores, and it was concluded that the percent change value had poor statistical efficiency for all correlations^35–37^. Adjusting for a baseline covariate can further improve the power of the comparisons and reduce the Intra-Class-Correlation-Coefficient which will improve the power^38^. We therefore fitted a multilevel model at T1 with cumulated defined daily doses (DDD) of PPI at T1 as the dependent variable, group as a predictor variable and cumulated defined daily doses (DDD) of PPI at T0 as a covariate. A multilevel model at T2 with cumulated defined daily doses (DDD) of PPI at T2 as the dependent variable, group as a predictor variable and cumulated defined daily doses (DDD) of PPI at T0 and T1 as covariates was also performed. Restricted Maximum Likelihood (REML) was used as estimation method. Adjusted means for the intervention and control groups were calculated and percentage reductions in the prescription of DDD of PPI were calculated.

We also calculated a longitudinal random intercepts model predicting cumulated defined daily doses (DDD) of PPI over time and using group membership as a predictor. We considered results with p values ≤ 0.05 to be significant. All analyses were performed with R version 4.0.2 and packages mice, broom.mixed, lme4, lmerTest, lmtest, mitml, emmeans, mosaic.

### Patient and public involvement

No patients or members of the public were involved in the design, analysis, interpretation or writing of the study. It was not the policy of the involved institutions to include patients in the planning or decision making processes at the time when the study was planned, submitted to ethical committees and funding agencies, and started.

## Results

### Participant flow

**Figure 1:**
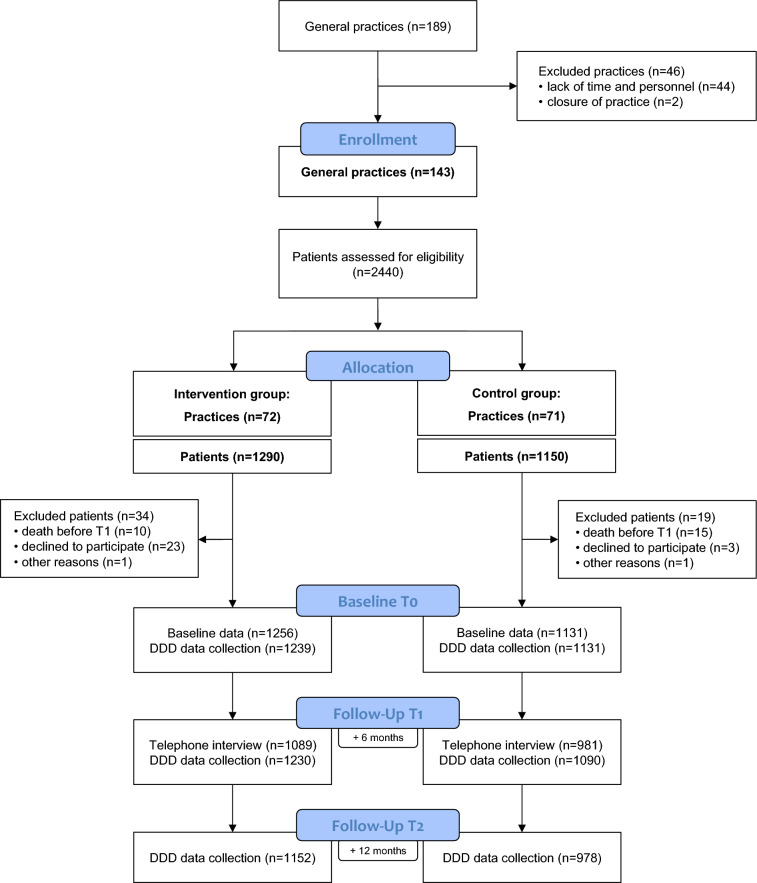
Flowchart of study participants.

In total, 2440 patients with a PPI medication of at least 6 months were recruited (Fig. 1). The patients were randomized on the practice level to the intervention or control group. After randomization, 53 patients were excluded from data collection because of death (n = 25), consent withdrawal (n = 26) or other reasons (n = 2; dementia, recruitment error). On average, 17 patients per practice were included in 143 practices. The intention-to-treat group comprised 1256 patients in the intervention group and 1131 patients in the control group with a ratio of 1.1 to 1. With regard to data collection, complete DDD data were available for 97.1% patients at T0, for 95.1% patients at T1, and for 87.3% at T2, and 84.4% of all patients were interviewed by phone at T1.

### Baseline data

Characteristics of GPs (n = 158) were well-balanced between intervention and control group regarding gender, age, practical experience, and practice location (Table 1). However, for general practices (n = 143), some differences appeared. In the intervention group, a slight predominance of group practices and less single practices occurred, but the effect size Cramér-V signaled a weak association^29^. Also, there was a trend towards a larger size of intervention practices compared to control practices of more medium size, but the effect size Cramér-V was moderate.

**Table 1:** Characteristics of general practices.

Characteristics	Intervention group (n = 72)	Control group (n = 71)	All (n = 143)	Statistical testing
General practitioner, n (%)^a^	80 (50.6%)	78 (49.4%)	158	
Gender, male, n (%)	55 (68.8%)	49 (62.8%)	104 (65.8%)	Chi-square p = .42
Age, years (means, SD)	54.00 (± 8.71)	53.89 (± 7.87)	53.95 (± 8.28)	t-Test p = .94
Practical experience, years (means, SD)	18.69 (± 10.36)	17.78 (± 10.19)	18.24 (± 10.25)	t-Test p = .75
Location				
Urban, n (%)	34 (47.2%)	38 (52.8%)	72	Chi-square p = 0.50 Cramér-V = 0.057
Rural, n (%)	36 (52.9%)	32 (47.2%)	68	
Type of practice				
Single practice, n (%)	29 (40.3%)	38 (54.3%)	67	Chi-square p = 0.10 Cramér-V = 0.14
Group practice, n (%)	43 (59.7%)	32 (45.7%)	75	
Practice size^b^				
Small	5 (6.9%)	6 (8.5%)	11	Chi-square p = 0.07 Cramér-V = 0.195
Medium	18 (25.0%)	30 (42.3%)	48	
Large	49 (68.1%)	35 (49.3%)	84	

^a^In some practices, more than one physician is recruited per practice (single-handed practices are still common in Germany).

^b^Practice size is determined by health insurance certificates per quarter; small: < 900 certificates, medium: 900–1500 certificates, large: > 1500 certificates.

At baseline, the recruited 2387 patients were 64.46 years of age (± 12.94) and 54.4% were female (Table 2). The most prevalent prescribed PPI agents were pantoprazole (64.4%) and omeprazole (29.7%) with an average DDD of 250 (± 8.0; independent of the PPI agent). The most common indications for PPI use were gastroesophageal reflux (41.4%) and gastroprotection as a preventive measure or together with NSAID/ASS (27.5%). More details are shown in Table 2. The characteristics between intervention and control group were well-balanced in terms of gender, age, prescribed PPI agent, defined daily dose (DDD) of PPI and indication for PPI uptake. The statistically significant differences which were observed are based predominantly on Chi-Square tests which are known to be dependent on sample size and they all have negligible effect sizes^29^.

**Table 2:** Characteristics of study patients.

Characteristics	Intervention group (n = 1256)	Control group (n = 1131)	All patients (n = 2387)	Statistical testing
Gender, female, n (%)	683 (54.4%)	615 (54.4%)	1298 (54.4%)	Chi-Square p = 0.99
Age, years (means, SE)	64.71 (± 12.71)	64.19 (± 13.19)	64.46 (± 12.94)	p = 0.373*
PPI agent, n (%)				
Pantoprazol	751 (66.2%)	646 (62.5%)	1397 (64.4%)	Fisher’s Exact Test, p = 0.18
Omeprazol	328 (28.7%)	316 (30.8%)	644 (29.7%)	
Esomeprazol	52 (4.6%)	66 (6.4%)	118 (5.4%)	
Lansoprazol	5 (0.4%)	4 (0.4%)	9 (0.4%)	
Dexlansoprazol	0 (0.0%)	0 (0.0%)	0 (0.0%)	
Rabeprazol	1 (0.1%)	0 (0.0%)	1 (0.05%)	
PPI DDD (means, SE)	256 (± 7.9)	244 (± 8.1)	250 (± 8.0)	p = 0.292*
Intake duration, years (means, SE)	5.46 (± 4.40)	5.14 (± 4.37)	5.30 (± 4.39)	p = 0.002, Cohen’s d = 0.19
Indication, n (%)				
Peptic ulcer	72 (7.0%)	78 (7.8%)	150 (7.4%)	Chi-square p = 0.48
Gastroesophageal reflux	508 (40.1%)	481 (42.5%)	989 (41.4)	Chi-square p = 0.66
Barrett's esophagus	97 (7.7%)	73 (6.5%)	170 (7.1%)	Chi-square p = 0.09
Chronic gastritis	168 (13.4%)	214 (18.9%)	382 (16.0%)	Chi-square p = 0.003, Cramér-V = 0.066
Gastroprotection	368 (29.3%)	289 (25.6%)	657 (27.5%)	Chi-square p = 0.001, Cramér-V = 0.072
Post hospital stay	64 (5.1%)	87 (7.7%)	151 (6.3%)	Chi-square p = 0.03, Cramér-V = 0.048
Unclear	25 (2.0%)	57 (5.0%)	82 (3.4%)	Chi-square p < 0.001, Cramér-V = 0.084

*t-test (Satterthwaite’s method).

### Consulting result in intervention group

After arriba-PPI training (T0), GPs of the intervention group consulted 1032 patients with arriba-PPI. In 33.4% of consultations (n = 419), GPs and patients agreed to discontinue PPI, in 15.8% of consultations (n = 199) they decided to reduce the dose. About one third of the patients did not change their medication (n = 390; 31.1%), in few cases the PPI agent was exchanged with another agent (n = 9; 0.7%) or the PPI dose was increased (n = 15; 1.2%).

### Primary outcome

The null model at T0 had an Intra-Class-Correlation Coefficient of 0.093 meaning that the correlation of cumulated defined daily doses (DDD) of PPI at T0 among patients within the same practices is about this value. Consequently, most of the variation in the outcome is among the lower-level units and therefore, correlation between them is relatively low^39^. After inclusion of the variable group as predictor there was no significant difference between the two groups at T0 regarding the DDD of PPI (p = 0.29). Means for cumulated defined daily doses (DDD) of PPI at T0 after inclusion of the cluster structure were 256 DDD (SE 7.86; 95% CI 240 to 271) for the intervention group and 244 DDD (SE 8.12; 95% CI 228 to 260) for the control group.

At T1 after the introduction of cumulated defined daily doses (DDD) of PPI at T0 as a covariate, the Intra-Class-Correlation Coefficient was reduced to 0.042. There was a significant difference between the two groups (p < 0.0001) in favour of the intervention group (Table 3). Means for cumulated defined daily doses (DDD) of PPI at T1 after considering cumulated defined daily doses (DDD) of PPI at T0 as a covariate were 199 DDD (SE 5.50; 95% CI 188–210) for the intervention group and 236 DDD (SE 5.95; 95% CI 224 to 248) for the control group.

**Table 3:** Results for the multilevel model at T1 with cumulated defined daily doses (DDD) of PPI at T0 as a covariate (ANCOVA model).

	Estimate	Standard error	Statistic	df	p
Intercept	61.43	7.20	8.54	2178.72	< 0.0001
control	37.16	8.15	4.56	1629.96	< 0.0001
DDD T0	0.55	0.02	30.25	1875.32	< 0.0001

**Figure 2:**
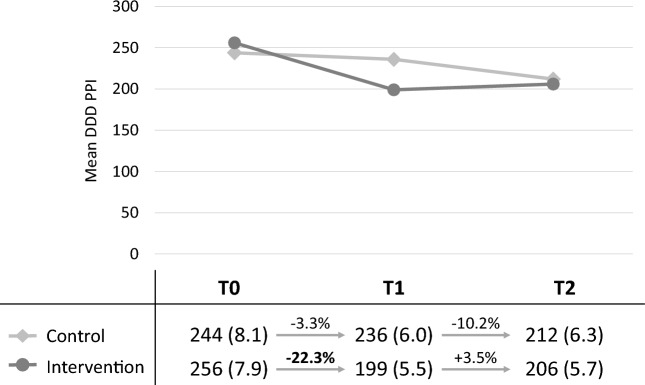
Course of the adjusted means (adjusted at T1 and T2 by the ANCOVA models) of the DDD of PPI in the intervention and control groups (average DDD with standard errors).

Compared to baseline (T0), there was a significant reduction in the PPI prescriptions after 6 months (T1) among study patients of intervention group (reduction of the mean PPI DDD: − 22.3% (95%CI − 18.55 to − 25.98), see Fig. 2. A reduction in PPI prescription of − 3.3% was observed in the control group (95% CI − 7.18 to + 0.62).

At T2 after the introduction of cumulated defined daily doses (DDD) of PPI at T0 and T1 as covariates, the Intra-Class-Correlation Coefficient was 0.05. There was no significant difference between the two groups (p = 0.48) (Table 4). Means for cumulated defined daily doses (DDD) of PPI at T2 after considering cumulated defined daily doses (DDD) of PPI at T0 and T1 as covariates were 206 DDD (SE 5.71; 95% CI 195 to 218) for the intervention group and 212 DDD (SE 6.26; 95% CI 200 to 225) for the control group.

**Table 4:** Results for the Multilevel model at T1 with cumulated defined daily doses (DDD) of PPI at T0 and T1 as covariates (ANCOVA model).

	Estimate	Std. error	Statistic	df	p
Intercept	65.09	7.62	8.54	643.64	< 0.0001
control	5.99	8.42	0.71	793.24	0.48
DDD T0	0.20	0.02	9.25	384.56	< 0.0001
DDD T1	0.41	0.02	17.88	213.34	< 0.0001

Follow-up at T2 showed that reduced DDD PPI remained stable in intervention patients with no further significant change compared to T1: + 3.5% (95%CI − 0.99 to + 8.03. Control patients showed a decrease in prescribed DDD PPI of -10.17% (95%CI − 6.01 to − 14.33) at T2 compared to T1.

The longitudinal random intercepts model confirmed the results of our multilevel ANCOVA models. The time effect was significant (estimate − 27.40, SE 3.36, p < 0.0001), as the number of DDD of PPI decreases over time. The group allocation did not show significance, as there was no significant difference between the intervention and control groups at both time T0 and time T2 (control, estimate − 1.32, SE 11.09, p = 0.91). The time*group interaction, on the other hand, was significant, as there was a differential trend with a significant difference at time T1 (estimate 15.82, SE 4.90, p = 0.001).

There were no relevant differences between the models with complete cases and with imputed data.

### PPI intake status over time

**Figure 3:**
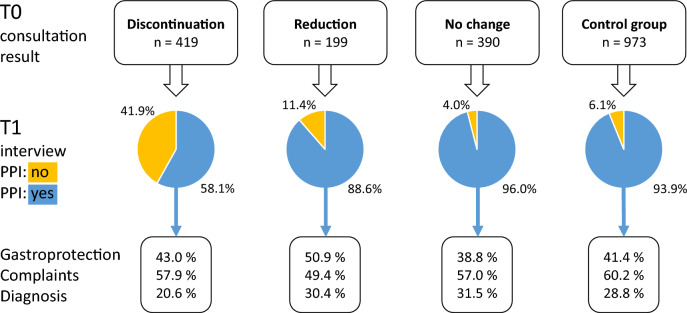
PPI intake status and reason for intake as reported by the patients themselves at T1, stratified by the results of the arriba-PPI consultation at T0.

Figure 3 shows the PPI intake status of patients over time according to the information provided by patients in the telephone interviews at T1. Among the patients in the intervention group who had decided to discontinue PPIs during their arriba-PPI based consultation at T0, 41.9% stayed on their course to stop taking PPIs 6 months later. More than half of these patients started taking PPIs again. Among participants who had decided for dose reduction during the consultation, 11.4% discontinued their PPI over the course of the study. Only few changes in PPI medication occurred among participants who had decided not to change their PPI medication at T0 and who were in the control group.

## Discussion

### Principal findings

In this study, the use of the discontinuation strategy arriba-PPI in general practice resulted in a greater reduction in PPI prescriptions than with usual care. Consultation with arriba-PPI led to discontinuation or reduction attempts in almost half the patients. More than a third of the patients who had decided to discontinue PPI are still taking no PPI at 6 months. Overall, DDD PPI prescription rates in all intervention patients were significantly decreased by 22% at 6 months and that level was maintained at 12 months.

### Discussion of the literature

Interventions that aim to change provider behavior to reach deprescribing of unnecessary PPI medication show similar outcomes. Lai et al.^18^ and Cateau et al.^40^ describe education sessions for physicians that range from 1h to half a day that show significant deprescribing results (47% success rate at 4 months and 13% at 1 year, respectively). These and our studies have an educational component in common. The physician has to give the impulse for a deprescribing conversation that also reflects his own beliefs, to be believable and trustworthy for the patient^41^. Publication of international guidelines is not sufficient and shows no or slight difference in prescribing patterns^17,42^. Rigid methods like a deprescribing algorithm or installing an Excel file with an embedded, automated scoring system show significant but lower success^19,20^.

Studies aiming at incorporating patients’ views and addressing their fears of poor symptomatic control, offer a self-management plan or provide measures for rebound problems and report 75%-83% stepping down or off PPI^43–45^. It should be noted that for these studies investigators prescreened their participants for inappropriate PPI use, whereas in our study we included all patients with long-term PPI use. arriba-PPI offers the possibility to decide whether a PPI is inappropriate. It seems crucial to include the patient in these deprescribing conversations to reach a common understanding^41^. Important topics to discuss are the necessity of PPI medication, the possibility of rebound symptoms, and symptom avoidance and control. It is essential for patients to understand why a PPI could be discontinued and for physicians to acknowledge their concern of recurring symptoms. Patient preferences are not always incorporated into PPI deprescribing decisions^46,47^. But some patients prefer to participate in a shared decision about deprescribing, including discussing their preferences, while other, mostly older patients, trust their physician to decide for them^48^. Discontinuation studies that deprescribe PPI without consultation or shared decision making, e.g. when there is no indication for PPI use after endoscopy, are also successful (27% or 15% off PPI after 1 year, without symptoms), although, patients with troublesome GERD presumably did not participate in the study^49,50^.

### How did the intervention work?

Apart from the software, our complex intervention included outreach visits (or “academic detailing”, AD^51^) with study team members visiting intervention group practices to deliver evidence-based information and educational contents about harmful effects of long-term PPI use and deprescribing. Chhina et al.^52^ showed in their review that AD can be effective at optimizing prescription of medications by GPs, albeit with overall moderate effect. The software arriba-PPI adds to the educational component by scaffolding new behavior and reinforcing self-efficacy of the GPs regarding PPI withdrawal. On one hand, clinicians and policymakers adopting decision support software should be aware that additional measures are required for implementation. On the other hand, implementation of a software can have effects beyond the use of the software itself. We report further insights from qualitative interviews with GPs and patients including a programme theory in a separate publication^53^.

### Barriers

In our study, most patients state complaints or gastroprotection and fewer self-reported diagnosis as reason for their PPI use at T1. This reflects the importance of symptomatic control. That makes PPI “notoriously hard to reduce” because they offer symptomatic relief in comparison to other PIMs like statins^40^.

Some GPs expect the stereotype of a patient demanding PPI prescriptions or of using PPI to support an unhealthy lifestyle^15^. However, many patients actually use PPI to maintain a healthier lifestyle, like eating fruit or drinking less alcohol. Patients are also more concerned about side-effects and the safety of PPIs than their doctors realized. Other studies show that a lot of patients (40–70%) are open-minded to discuss deprescribing and would like to take the lowest effective dose^16^. Patients understand the rationale for deprescribing and appreciate receiving specific advice on a deprescribing plan. Biggest concern is the possibility of symptom returning, 68% of patients do not condone the return of any symptoms, even minor ones, but patients are encouraged if they know they can restart their PPI medication if necessary. Prolonged reflux symptoms on PPI therapy are associated with reduced physical and mental quality of life^54^.

Gastroprotection with PPI during NSAID therapy may become relevant in patients older than 60 years and with risk factors^55^. Even in those groups, the risk of peptic ulcer disease (PUD) is not sufficiently high to warrant gastroprotection to everyone. Patients starting long-term NSAID or ASA therapy should be tested for *H. pylori* first to start eradication treatment; this will sufficiently reduce PUD complications^55^. Additional risk factors can be determined by a scoring system (Table 1^55^).

### Strengths and limitations of the study

This study had numerous strengths: To counter selection bias at recruitment, patients were asked systematically and consecutively in the general practice when picking up their refill prescription for PPI. Medical assistants proposed to participate in a study to discuss medication status of their stomach medication with their physician; a possible discontinuation was not yet addressed at this point.

By selecting three study sites across federal state lines, the results were not localized and possible differences in PPI prescription patterns in different states were compensated. Furthermore, the German health insurance company BARMER identified high prescribing practices in Hessen and Westphalia and invited the top 13.8% of the PPI prescribing practices to participate in our study. At least 10 out of 143 study practices were high prescribers. The sample of practices was thus highly generalizable. Although this was a cluster-randomized study, relevant characteristics of participating patients and practices were well balanced between study arms.

For studies of this kind, practices interested in the topic are easier to motivate to take part. Since these have often reflected on their prescribing behavior, ceiling effects may result. Despite this, the implementation of arriba-PPI was effective at T1.

Between the 6th and 12th month of data collection, there was also a decrease in PPI prescriptions in the control group, so that the difference between the intervention and control group was no longer significant at the end of the data collection period. Measures in the health care system were introduced to reduce PPI prescribing. This included not only information on risks and cost, but also threatened prescribing GPs with monetary sanctions for inappropriate prescribing. This could have resulted in a more conscious way to prescribe PPI.

We also cannot exclude a certain degree of contamination. The patient interview at 6 months may have influenced the control group’s attitude toward PPI use. As there was no consultation planned in this group, the phone interview was the only conversation for these patients concerning their medication. Also there could be a certain degree of social desirability bias when patients were directly asked about their PPI uptake. Lastly, interviewers were not blinded.

### Clinical impact and future research

arriba-PPI is an effective tool that can also be used as a source of personal training. Even when not applied in daily consultations, the physician is attuned to PPI overuse and can give his patients an impulse to change their way of thinking. As most of the conservations with patients are centered on planning and follow-up, information and training should happen beforehand^56^. Patient-specific deprescribing interventions lead to less medication prescriptions than interventions that target institutions, like whole general practices or nursing homes^57^. Future research should focus on individualized approaches to avoid inappropriate medication use, training of the physician and possibilities for implementation.

## Data Availability

Pseudonymized data are available on reasonable request from the corresponding author (julia.heisig@uni-marburg.de) on approval and with a signed data access agreement.

## Abbreviations

CI: Confidence interval
DDD: Defined daily dose
GERD: Gastroesophageal reflux disease
GP: General practitioner
NSAID: Nonsteroidal anti-inflammatory drugs
PPI: Proton pump inhibitor
PUD: Peptic ulcer disease
SE: Standard error

## References

[1] Katz PO, Gerson LB, Vela MF (2013). Guidelines for the diagnosis and management of gastroesophageal reflux disease. Am. J. Gastroenterol..

[2] Koop H (2014). S2k-Leitlinie. Gastroösophageale Refluxkrankkheit unter Federführung der Deutschen Gesellschaft für Gastroenterologie, Verdauungs- und Stoffwechselkrankheiten (DGVS):AWMF Register Nr. 021-013. Zeitschrift fur Gastroenterologie.

[3] Fischbach W (2017). S2k-Leitlinie Helicobacter pylori und gastroduodenale Ulkuskrankheit. Z. Gastroenterol..

[4] Freedberg DE, Kim LS, Yang Y-X (2017). The risks and benefits of long-term use of proton pump inhibitors. Expert review and best practice advice from the American Gastroenterological Association. Gastroenterology.

[5] Savarino V, Di Mario F, Scarpignato C (2009). Proton pump inhibitors in GORD: An overview of their pharmacology, efficacy and safety. Pharmacol. Res..

[6] Yang Y-X, Metz DC (2010). Safety of proton pump inhibitor exposure. Gastroenterology.

[7] Bell EJ (2021). Association of proton pump inhibitors with higher risk of cardiovascular disease and heart failure. Mayo Clin. Proc..

[8] Parente F (2003). Hospital use of acid-suppressive medications and its fall-out on prescribing in general practice. A 1-month survey. Aliment. Pharmacol. Ther..

[9] Grimmsmann T, Schwabe U, Himmel W (2007). The influence of hospitalisation on drug prescription in primary care—A large-scale follow-up study. Eur. J. Clin. Pharmacol..

[10] Naunton M, Peterson GM, Bleasel MD (2000). Overuse of proton pump inhibitors. J. Clin. Pharm. Ther..

[11] Molloy D, Molloy A, O'Loughlin C, Falconer M, Hennessy M (2010). Inappropriate use of proton pump inhibitors. Irish J. Med. Sci..

[12] Clarke K (2021). Reducing overuse of proton pump inhibitors for stress ulcer prophylaxis and nonvariceal gastrointestinal bleeding in the hospital. A narrative review and implementation guide. J. Hosp. Med..

[13] Rückert-Eheberg I-M (2021). Who gets prescriptions for proton pump inhibitors and why? A drug-utilization study with claims data in Bavaria, Germany, 2010–2018. Eur. J. Clin. Pharmacol..

[14] Wermeling M, Himmel W, Behrens G, Ahrens D (2014). Why do GPs continue inappropriate hospital prescriptions of proton pump inhibitors? A qualitative study. Eur. J. Gen. Pract..

[15] Grime J, Pollock K, Blenkinsopp A (2001). Proton pump inhibitors. Perspectives of patients and their GPs. Br. J. Gen. Pract..

[16] Thompson W (2018). Patient values and preferences surrounding proton pump inhibitor use. A scoping review. Patient.

[17] Abrahami D, McDonald EG, Schnitzer M, Azoulay L (2021). Trends in prescribing patterns of proton pump inhibitors surrounding new guidelines. Ann. Epidemiol..

[18] Lai A, Odom A, Roskos SE, Phillips JP (2021). Deprescribing inappropriate proton pump inhibitors in a family medicine residency practice office. PRiMER.

[19] Kastner M (2021). Choosing wisely. An idea worth sustaining. Health Serv. Res..

[20] Calvo LLJ (2021). Successful deprescribing of proton pump inhibitors with a patient-centered process. The DESPIBP Project. Eur. J. Clin. Pharmacol..

[21] Wilsdon TD, Hendrix I, Thynne TRJ, Mangoni AA (2017). Effectiveness of interventions to deprescribe inappropriate proton pump inhibitors in older adults. Drugs Aging.

[22] Helgadottir H, Bjornsson ES (2019). Problems associated with deprescribing of proton pump inhibitors. Int. J. Mol. Sci..

[23] Hirsch O, Keller H, Krones T, Donner-Banzhoff N (2011). Acceptance of shared decision making with reference to an electronic library of decision aids (arriba-lib) and its association to decision making in patients. An evaluation study. Implement. Sci..

[24] Rieckert A (2019). Reduction of the long-term use of proton pump inhibitors by a patient-oriented electronic decision support tool (arriba-PPI). Study protocol for a randomized controlled trial. Trials.

[25] Haastrup PF (2021). When does proton pump inhibitor treatment become long term? A scoping review. BMJ Open Gastroenterol..

[26] Daniels B, Pearson S-A, Buckley NA, Bruno C, Zoega H (2020). Long-term use of proton-pump inhibitors: Whole-of-population patterns in Australia 2013–2016. Ther. Adv. Gastroenterol..

[27] Farrell B (2017). Deprescribing proton pump inhibitors. Evidence-based clinical practice guideline. Can. Fam. Phys. Medecin de famille canadien.

[28] Fricke, U., Günther, J., Niepraschik-von Dollen, K. & Zawinell, A. Anatomisch-therapeutisch-chemische Klassifikation mit Tagesdosen für den deutschen Arzneimittelmarkt. ATC-Index mit DDD-Angaben. (2022).

[29] Kotrlik, J. W. & Williams, H. A. The incorporation of effect size in information technology, learning, and performance research. *Inf. Technol. Learn. Perform. J.* **21**(1), 1–7 (2003).

[30] van Buuren S (2018). Flexible Imputation of Missing Data.

[31] Pedersen AB (2017). Missing data and multiple imputation in clinical epidemiological research. Clin. Epidemiol..

[32] Sterne JAC (2009). Multiple imputation for missing data in epidemiological and clinical research, Potential and pitfalls. BMJ.

[33] Finch WH, Bolin JH, Kelley K (2019). Multilevel modeling using R.

[34] Norman GR (1989). Issues in the use of change scores in randomized trials. J. Clin. Epidemiol..

[35] Vickers AJ (2001). The use of percentage change from baseline as an outcome in a controlled trial is statistically inefficient. A simulation study. BMC Med. Res. Methodol..

[36] Vickers AJ, Altman DG (2001). Statistics notes. Analysing controlled trials with baseline and follow up measurements. BMJ.

[37] Clifton L, Clifton DA (2019). The correlation between baseline score and post-intervention score, and its implications for statistical analysis. Trials.

[38] Campbell MJ, Walters SJ (2014). How to Design, Analyse and Report Cluster Randomised Trials in Medicine and Health Related Research.

[39] Monsalves MJ, Bangdiwala AS, Thabane A, Bangdiwala SI (2020). LEVEL (logical explanations & visualizations of estimates in linear mixed models). Recommendations for reporting multilevel data and analyses. BMC Med. Res. Methodol..

[40] Cateau D, Ballabeni P, Niquille A (2021). Effects of an interprofessional Quality Circle-Deprescribing Module (QC-DeMo) in Swiss nursing homes. A randomised controlled trial. BMC Geriatr..

[41] Thompson W (2020). Discussing proton pump inhibitor deprescribing. The views of Danish GPs and older patients. BMC Fam. Pract..

[42] Farrell B (2018). Self-efficacy for deprescribing. A survey for health care professionals using evidence-based deprescribing guidelines. Res. Soc. Adm. Pharm..

[43] Ayoub J, McGregor JC, Castner RM, Singh H (2021). Opportunities for successful de-escalation of proton pump inhibitors at a federally qualified health center. BMC Pharmacol. Toxicol..

[44] Coyle C (2019). Sustained proton pump inhibitor deprescribing among dyspeptic patients in general practice. A return to self-management through a programme of education and alginate rescue therapy. A prospective interventional study. BJGP Open.

[45] Murie J, Allen J, Simmonds R, de Wet C (2012). Glad you brought it up A patient-centred programme to reduce proton-pump inhibitor prescribing in general medical practice. Qual. Prim. Care.

[46] Mangin D (2019). 'I think this medicine actually killed my wife'. Patient and family perspectives on shared decision-making to optimize medications and safety. Ther. Adv. Drug Saf..

[47] Muscat DM (2019). Discussions about evidence and preferences in real-life general practice consultations with older patients. Patient Educ. Counsel..

[48] Weir K (2018). Decision-making preferences and deprescribing. Perspectives of older adults and companions about their medicines. J. Gerontol. Ser. B. Psychol. Sci. Soc. Sci..

[49] Björnsson E (2006). Discontinuation of proton pump inhibitors in patients on long-term therapy. A double-blind, placebo-controlled trial. Aliment. Pharmacol. Ther..

[50] Inadomi JM (2001). Step-down management of gastroesophageal reflux disease. Gastroenterology.

[51] Soumerai SB (1990). Principles of educational outreach ('academic detailing') to improve clinical decision making. JAMA.

[52] Chhina HK (2013). Effectiveness of academic detailing to optimize medication prescribing behaviour of family physicians. J. Pharm. Pharm. Sci..

[53] Schmidt A (2023). Patients' perspectives on a patient-oriented electronic decision support tool to reduce overuse of proton pump inhibitors (arriba-PPI): A qualitative study in primary care. BMC Prim. Care.

[54] Becher A, El-Serag H (2011). Systematic review. The association between symptomatic response to proton pump inhibitors and health-related quality of life in patients with gastro-oesophageal reflux disease. Aliment. Pharmacol. Ther..

[55] Kanno, T. & Moayyedi, P. Who needs gastroprotection in 2020? *Curr. Treat. Options Gastroenterol.* **18**, 557–573 (2020). 10.1007/s11938-020-00316-9 PMC7656506 33199955

[56] Turner JP (2018). Deprescribing conversations. A closer look at prescriber-patient communication. Ther. Adv. Drug Saf..

[57] Page AT, Clifford RM, Potter K, Schwartz D, Etherton-Beer CD (2016). The feasibility and effect of deprescribing in older adults on mortality and health. A systematic review and meta-analysis. Br. J. Clin. Pharmacol..

